# Captive Reptile Mortality Rates in the Home and Implications for the Wildlife Trade

**DOI:** 10.1371/journal.pone.0141460

**Published:** 2015-11-10

**Authors:** Janine E. Robinson, Freya A. V. St. John, Richard A. Griffiths, David L. Roberts

**Affiliations:** Durrell Institute of Conservation and Ecology, School of Anthropology and Conservation, University of Kent, Canterbury, Kent, CT2 7NR, United Kingdom; GI Lab, UNITED STATES

## Abstract

The trade in wildlife and keeping of exotic pets is subject to varying levels of national and international regulation and is a topic often attracting controversy. Reptiles are popular exotic pets and comprise a substantial component of the live animal trade. High mortality of traded animals raises welfare concerns, and also has implications for conservation if collection from the wild is required to meet demand. Mortality of reptiles can occur at any stage of the trade chain from collector to consumer. However, there is limited information on mortality rates of reptiles across trade chains, particularly amongst final consumers in the home. We investigated mortality rates of reptiles amongst consumers using a specialised technique for asking sensitive questions, additive Randomised Response Technique (aRRT), as well as direct questioning (DQ). Overall, 3.6% of snakes, chelonians and lizards died within one year of acquisition. Boas and pythons had the lowest reported mortality rates of 1.9% and chameleons had the highest at 28.2%. More than 97% of snakes, 87% of lizards and 69% of chelonians acquired by respondents over five years were reported to be captive bred and results suggest that mortality rates may be lowest for captive bred individuals. Estimates of mortality from aRRT and DQ did not differ significantly which is in line with our findings that respondents did not find questions about reptile mortality to be sensitive. This research suggests that captive reptile mortality in the home is rather low, and identifies those taxa where further effort could be made to reduce mortality rates.

## Introduction

The global legal trade in live animals (including primates, cage birds, birds of prey, reptiles and ornamental fish) was estimated to be worth €406 million in 2005, involving hundreds of millions of animals [1]. Reptiles are popular exotic pets [2, 3], and comprise an estimated 21% of the value of the live animal trade, excluding ornamental fish [1]. Reptiles entering trade are sourced directly from the wild, or are captive bred, with a large number produced in private and commercial breeding operations within consumer countries [4, 5, 6]. In the UK, the reptile sector of the pet industry alone is estimated to be worth £200 million, with approximately 250,000 reptiles and amphibians bred each year [4]. Due to concerns raised regarding biodiversity loss [7, 8], environmental, human and animal health [9–11], animal welfare [12] and also ethical and moral considerations [13], the trade attracts debate between reptile keepers, conservationists, veterinarians, animal welfare and animal protection groups.

The trading and keeping of exotic pets is subject to varying degrees of regulation, from international legislation such as the Convention on International Trade in Endangered Species (CITES) to national and regional legislation, including highly regulated (e.g. Australia, New Zealand, Norway), or a largely permitted trade with only certain species prohibited (e.g. European Union [14]). The trade is also influenced by a range of different environmental policy debates, for example, US regulations were recently amended to add large constrictor snakes to the Species Listed as Injurious Wildlife under the Lacey Act, on the grounds of ecosystem damage [15], whilst recent discussions in the EU have concerned EU legislation on Invasive Alien Species. In Norway, the keeping of exotic animals is prohibited under the Animal Welfare Act and despite attempts to open trade in a limited number of reptile and amphibian species, in 2013 this was rejected by the Norwegian government amidst opposition from groups opposed to the trade [16, 17]. Additionally, in early 2015, Scotland announced plans to review exotic pet keeping legislation, following discussions with animal welfare charities [18].

Animals may die during any part of the trade chain, from collection in the wild, in transit, or in the home. This not only raises animal welfare concerns, but can also have conservation implications if animals are unsustainably sourced from the wild. However, there are few data in peer-reviewed literature concerning mortality of reptiles in the home (i.e. in the hands of an end-consumer). Indeed, much of the research regarding traded reptile mortality is outdated [19–21], in grey literature [22], concentrated on isolated cases [23] and considers alternative locations along the supply chain other than ‘the home’. For example, the most comprehensive study to date concerning mortality in transit, analysed data for more than 7.4 million individual animals and reported an average dead on arrival (DOA) mortality rate of 3.14% for reptiles [24]. Additionally, of around 3,000 reptile shipments, less than 1% had mortality of over 50% DOA, whilst 72.7% had zero mortality [24]. Previous studies have also revealed low levels of mortality; less than 0.5% of 8,000 reptiles and amphibians coming into the UK died in transit [25]; and less than 1% of tortoises transported over 21 years from the Mediterranean to the UK via air transport and long distance lorries were DOA or dead within a week of delivery [19]. In retail, mortality rates ranging from 1.69 to 4.4% in shops prior to sale have been reported [20, 25]. Whilst these results suggest that typical mortality rates at the transport and retail stages of the chain are relatively low, there have been reported examples of much higher losses. Such incidents often concern isolated cases but they are typically the ones that receive considerable media attention. For example, 400 reptiles and amphibians from Madagascar died in transit in South Africa [26, 27] due to flight delays following bad weather [27], and a mortality rate of 72% was reported during a six week stock turnover period in one wildlife wholesaler [23].

The limited studies regarding mortality rates after purchase from pet shops report vastly different mortality rates. For example, Lawrence [21] reported annual mortality rates of 23% and 29%, between 1982 and 1986, for Spur-thighed (*Testudo graeca*) and Hermann’s tortoises (*T*. *hermanni*), respectively. These mortality rates were attributed to problems associated with hibernation [21] as well as low pricing and consequent use as pets for children [28]. Additionally, the composition of tortoises in trade is likely to have changed significantly since this time following an EU wide ban on wild-caught Mediterranean tortoises implemented in 1984 through EU Wildlife Trade Regulations (Council Regulation 3626/82). A more recent study using online questionnaires with over 800 respondents reported an annual reptile mortality rate of 3.25% [22]. In contrast, a much higher mortality of over 75% was obtained based on the difference between the estimated number of reptiles coming into the UK and the estimated number in the home [29]. Given the lack of published studies and widely conflicting available reports, it is evident that current primary data on mortality rates of reptiles in the home would be welcomed by all interested stakeholders.

Obtaining data on mortality of reptiles in the home relies on gathering information from consumers. However, given the potential sensitivity of issues surrounding the exotic reptile trade, estimating mortality rates using a conventional questionnaire may be problematic and prone to a number of biases [30]. Two such biases are social-desirability bias and non-response bias. Social-desirability bias occurs when respondents provide dishonest answers to present themselves in a more favourable manner relative to existing social norms [31]. Non-response bias results from a non-random and significant proportion of individuals refusing to take part in a survey [32]. Specialised questioning techniques have been developed within the social sciences to help improve the validity of sensitive data. These techniques work by ensuring respondents’ answers cannot be linked to them directly, even when questions are delivered via face-to-face interviews, thereby increasing the level of protection afforded to respondents and their willingness to answer honestly [30]. The Randomized Response Technique (RRT) [33] is one such specialised technique, which has been shown to significantly improve the validity of data when investigating sensitive or illegal behaviours [34, 35].

We investigated mortality rates of pet reptiles amongst domestic reptile keepers at two major herpetological events in the UK, using both direct questions (DQ) and additive RRT (aRRT). Specifically, we addressed the following questions: (1) What proportion of reptiles die within one year of acquisition? (2) Which commonly kept reptile groups are most susceptible to dying within one year of acquisition? (3) Are captive bred or wild caught reptiles more likely to die within one year of acquisition? The findings are intended to inform the ongoing debate concerning the regulation of the reptile trade and help safeguard species threatened by international commerce.

## Methods

### Data collection

A questionnaire (S1 File) was administered through face-to-face interviews by a team of six to 10 trained research assistants, at two major herpetological events in the UK: the Federation of British Herpetologists Accredited Breeders Meeting at Kempton Park (London) in August 2013, and the International Herpetological Society’s Breeders Meeting at Doncaster Racecourse in September 2013. Survey work was conducted with permission from event organisers. Both meetings attract between 2,000 and 5,000 visitors annually. Non-probability convenience sampling [36] was used to select respondents entering the venue (whilst queueing for entry), and within the venue, making use of breakout areas (e.g. cafeteria) to approach respondents. Names and contact details were not collected in order to assure anonymity. Only respondents who had acquired a reptile in the preceding five years were interviewed in order to minimise recall bias. By ‘acquired’ we refer to reptiles brought into the respondent’s home via purchase, gifting or loan, but excluding animals bred by the respondent. These were excluded in order to avoid juvenile mortality during breeding biasing results.

The survey consisted of a series of questions relating to: reptile ownership; reptile mortality rates experienced by respondents; number of years keeping reptiles; demographic questions (e.g. age, gender, area of residence), and questions designed to explore the sensitivity of the topic and evaluate the aRRT methodology. Questions concerning reptile ownership and mortality initially focussed on three reptile groups: snakes, chelonians (tortoises and turtles), and lizards, and then focussed on more specific categorisation of reptile groups. For snakes, this included: ‘boas and pythons’, ‘king and rat snakes’ and ‘other snakes’; for chelonians this included: ‘tortoises and box turtles’ and ‘turtles and terrapins’; and for lizards: ‘chameleons’, ‘geckos’, ‘skinks’, ‘iguanas’, ‘tegus and monitors’, ‘agamids’ and ‘other lizards’. Respondents were asked to indicate whether the reptiles they had acquired were captive bred, wild, captive-farmed, or of unknown source. According to CITES, captive bred refers to animals bred in a controlled environment to second generation or beyond, and captive farmed or ‘ranched’ usually refers to reptiles reared in countries where the species naturally occur, either from young or eggs collected in the wild, or from wild collected pregnant/gravid females [37].

To investigate mortality rates, respondents were asked the following questions using aRRT and DQ with ‘X’ representing each reptile group the respondent had acquired: “Of the X that you acquired over the last five years, how many died within 12 months of acquisition”. Following this, respondents were asked how many individuals of each reptile group they had acquired in the preceding five years. To understand how people perceived difficulty and survival of their reptiles in captivity, respondents were asked, based on their own experience and not preconceived ideas about the reptile group, to rate the difficulty in keeping each of the reptile groups they had owned, and to rate the survival of each group in captivity, according to a five point Likert scale. All respondents were asked the average amount of time that they kept their reptiles for in order to exclude any that sold or exchanged their animals within a year.

### Additive Randomized Response Technique

All forms of RRT use a randomizing device, such as a deck of cards or dice, to scramble respondents’ answers to sensitive questions. This increases respondent privacy and ensures that researchers cannot directly link answers to individuals. However, the aggregate proportion of people holding the sensitive characteristic can be estimated using probability theorem [30, 33]. RRT typically estimates the proportion of the study population holding the stigmatizing characteristic, yet we often want to understand the quantitative nature of sensitive acts [30]. Additive RRT [38] can be used when quantitative responses are required, rather than binary (yes-no) responses. Our aRRT followed a ‘partial’ (two-stage) quantitative randomization model [39], whereby a proportion of respondents were instructed to answer the sensitive question truthfully and a proportion were asked to add a number to their true response based on a randomization device. The randomization device consisted of a standard deck of playing cards, including four Queens but excluding Jacks and Kings, therefore comprising a total of 44 cards. If the respondent picked a Queen (probability = 0.09) they were instructed to answer the question about the number of reptiles that had died truthfully. If the respondent picked any number card (probability = 0.91), they were instructed to add the number on the card to their true response and report the sum (e.g. seven hearts + two dead reptiles = nine). Respondents were instructed not to reveal their selected card to their interviewer, as such, interviewers could not distinguish truthful responses from scrambled ones; they simply recorded a number. However, as the numbers in the deck followed a known probability distribution and the mean and variance of the number cards was known, a mean value for the true responses could be calculated using the following formula:

$$\mu_{x}=Y-(1-T)\mu_{s}$$

where T is the proportion of cards asking respondents to answer truthfully, Y is the reported response, X is the true sensitive variable of interest with unknown mean μ_x_ and unknown variance σ^2^_x_, and S is the scrambling variable with known true mean μ_s_ and known variance σ^2^_s_ [39]

Additive RRT was explained to respondents using a simple example and they were asked to follow the method carefully so that their answers were scrambled and the data were not compromised. An instruction card (S2 File) was also handed to the respondent stating: “Queen–answer the question truthfully, number card–add the number on the card you have picked to your true response and report the total” and reminded them of the question: “Of the X that you acquired over the last five years, how many died within the first 12 months?” The questionnaire commenced once the interviewer was satisfied that the respondent understood the method. See S3 File for additional information regarding the aRRT methodology.

### Direct questions

In order to explore the relative utility of aRRT compared to conventional DQ, at the end of the questionnaire, respondents were asked to directly answer the same questions asked previously using aRRT, this time not using the cards: “Of the X that you acquired over the last five years, how many died within 12 months of acquisition”.

### Data analysis

The mean number of reptiles that died within one year of acquisition and associated 95% confidence intervals were calculated from 1,000 samples [34] bootstrapped by respondent identification number for both aRRT and DQ responses, in the former case, incorporating the above formula to calculate the true responses from reported responses. We considered that there was no significant difference between estimates achieved via aRRT and DQ when the bootstrapped 95% confidence intervals for the mean number of reptiles dying overlapped with each other. Subsequently, mean mortality rates (i.e. the *proportion* of respondents’ reptiles that died within a year) along with 95% confidence intervals were generated by incorporating the number of reptiles acquired over the previous five-year period into the bootstrap.

Spearman’s rank correlations were used to investigate the relationship between mortality rates obtained by DQ, and: respondents’ opinions regarding how sensitive they thought the questions about reptile mortality were and how likely respondents’ thought people would be to tell the truth about their reptiles dying. Spearman’s Rank correlations were also used to explore the relationship between reported mortality rates, and how respondents rated survival and difficulty level for different reptile groups.

Ethical approval was granted by the School of Anthropology and Conservation Research Ethics Advisory Group (University of Kent). Written consent was obtained from all respondents prior to interview by means of a tick box on the questionnaire and persons under 18 were not interviewed. Data were analysed using R v3.0.1 (R Development Core Team, 2014).

## Results

Two hundred and sixty five questionnaires (91 from Kempton Park and 174 from Doncaster) were completed by private keepers and breeders of reptiles, owning a total of 6,689 reptiles. Data from four commercial operations were analysed separately. Three respondents were excluded from the analysis as they refused to follow aRRT instructions. Respondents ranged in age from 18 to 72 years (median = 32, interquartile = 19, n = 255) and 72% of respondents were male (n = 189). Respondents came from all over the UK residing in 74% of the 121 recognized postcode areas in the UK.

Individual respondents reported keeping between 1 and 1,003 snakes (median = 9, interquartile = 20, n = 203), 1 and 30 chelonians (median = 2, interquartile = 3, n = 62) and 1 and 60 lizards (median = 5, interquartile = 6, n = 185) over the five-year period preceding the study. The total time respondents’ had kept reptiles varied with 9% (n = 24) having kept reptiles for less than one year, 21% (n = 54) for 2–5 years, 24% (n = 62) for 6–10 years, 26% (n = 67) for 11–20 years and 20% (n = 53) for 21 years or more. Thirty six percent (n = 32, asked at Kempton Park only) of respondents belonged to a herpetological group or society, including the International Herpetological Society (IHS), British Herpetological Society (BHS), a local or regional herpetological society (e.g. Thames & Chiltern Herpetological Group), or any other taxa specific (e.g. British Chelonian Group), herpetological or conservation society (e.g. Amphibian and Reptile Groups ARG UK).

Over 97% of snakes, 69% of chelonians and 87% of lizards acquired by respondents over the preceding five years were reported to be captive bred (Table 1).

**Table 1:** Percentage of reptiles acquired over five years preceding the study which were reported by respondents (N = 265) to be captive bred, wild, captive farmed or of unknown origin. Also includes the number of respondents and the total number of individual animals used in the analysis.

Taxa	% captive bred	% wild	% captive farmed	% unknown	*n* (respondents)	*n* (animals)^a^
**All snakes**	97.1	1.2	0.8	0.4	203	4954
Boas & pythons	96.2	0.8	1.1	0.1	165	3517
King & rat snakes	97.4	0.8	0.0	1.6	134	1038
Other snakes	92.3	2.6	0.0	0.5	55	417
**All chelonians**	69.2	9.1	5.1	12.3	62	276
Tortoises & box turtles	70.9	9.1	9.1	10.9	49	165
Turtles & terrapins	48.4	9.9	0.0	25.3	18	91
**All lizards**	86.8	6.3	2.1	2.3	185	1459
Chameleons	88.8	3.1	1.0	5.1	39	98
Geckoes	93.2	2.8	0.0	2.9	120	782
Skinks	83.3	11.1	0.0	2.8	17	36
Iguanas	76.1	15.2	2.2	6.5	22	46
Tegus & monitors	68.3	9.9	12.9	2.0	43	101
Agamids	84.1	7.8	0.0	2.6	84	271
Other lizards	58.6	20.0	0.0	0.0	20	70

^a^ Some respondents’ were unable to provide data for the more detailed categories e.g. ‘boas and pythons’, therefore their sum is not always equal to the total for that group e.g. ‘all snakes’. The total number of reptiles used in the study is calculated from the sum of the ‘all snakes’, ‘all chelonians’ and ‘all lizards’ categories.

### Mortality rates

There were no significant differences between the mean number of reptile deaths reported via aRRT and DQ for all taxonomic groups (Table 2) suggesting that respondents were generally amenable to reporting directly (i.e. via DQ) the quantity of reptiles that had died in their care. As aRRT did not appear to increase data validity (e.g. an increase in honest reporting indicated by estimates significantly higher than DQ) mortality rates obtained via DQ are used for the remaining analyses.

**Table 2:** Bootstrapped mean number of reptiles that died within a year of acquisition, over five years preceding the study, including 95% confidence intervals, estimated for additive (aRRT) and direct questions (DQ) via 1000 bootstrap samples.

		aRRT			DQ		
Taxa	*n*	Mean no. reptiles that died	lower CI	upper CI	Mean no. reptiles that died	lower CI	upper CI
**All reptiles** ^a^	256	NA	NA	NA	0.89	0.62	1.17
**All snakes**	201	0.35	-0.13	0.83	0.55	0.37	0.72
Boas & pythons	163	0.06	-0.49	0.61	0.28	0.16	0.41
King & rat snakes	132	0.21	-0.36	0.79	0.40	0.17	0.63
Other snakes	53	0.33	-0.57	1.23	0.35	0.08	0.61
**All chelonians**	62	0.54	-0.30	1.39	0.17	-0.01	0.34
Tortoises & box turtles	49	0.54	-0.47	1.54	0.07	-0.01	0.14
Turtles & terrapins	18	0.60	-0.89	2.09	0.38	-0.15	0.92
**All lizards**	178	0.21	-0.31	0.73	0.66	0.38	0.94
Chameleons	36	0.47	-0.77	1.72	0.74	0.03	1.45
Geckoes	115	0.21	-0.45	0.87	0.39	0.26	0.51
Skinks	17	0.49	-1.35	2.34	0.20	-0.08	0.50
Iguanas	22	0.62	-1.11	2.35	0.10	-0.04	0.23
Tegus & monitors	41	-0.19	-1.26	0.88	0.21	0.03	0.39
Agamids	78	0.46	-0.27	1.19	0.23	0.08	0.39
Other lizards	19	0.18	-1.45	1.81	0.00	0.00	0.00

**Fig 1:**
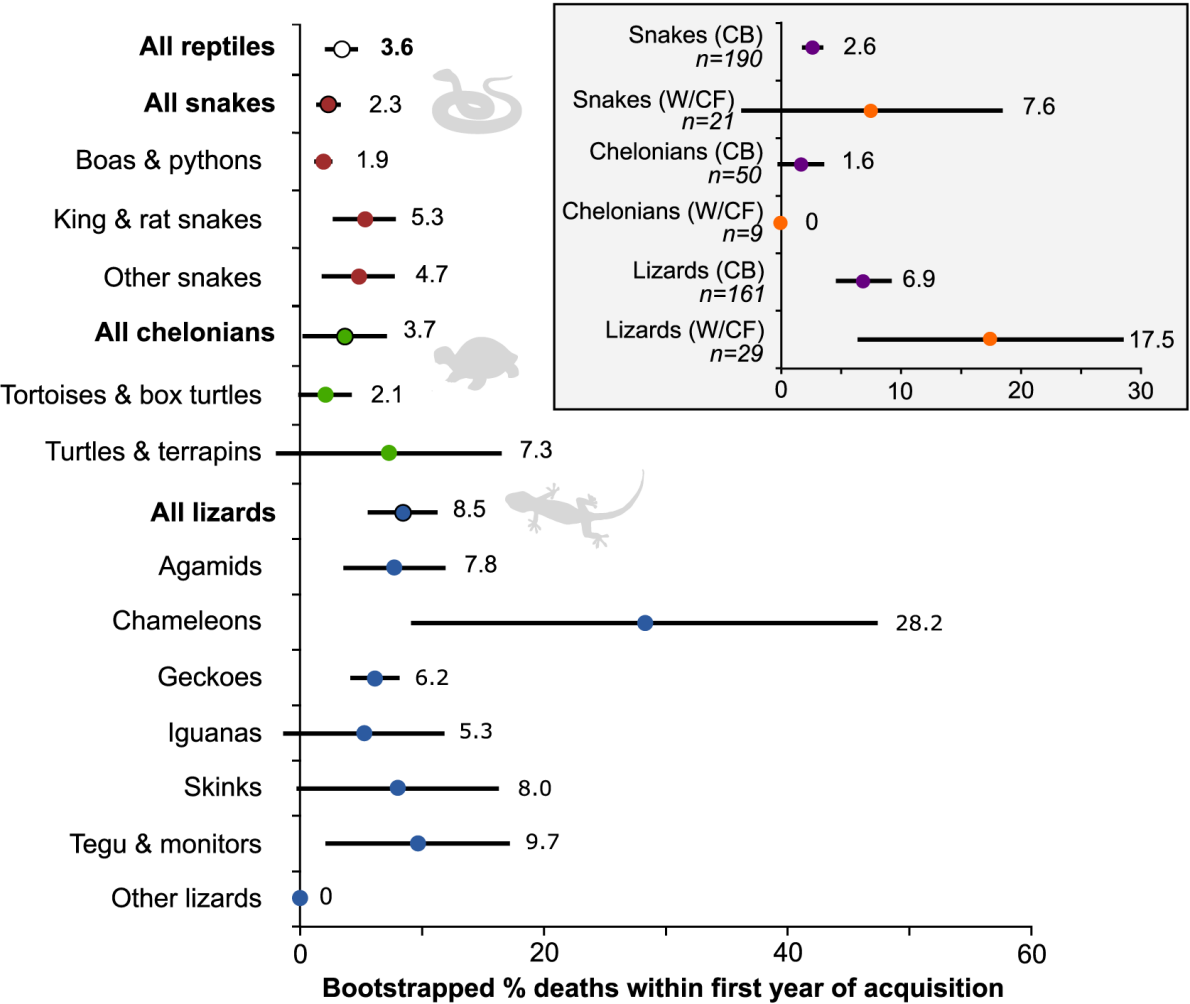
Bootstrapped reptile mortality rates within first year of acquisition. The bootstrapped proportion of deaths within first year of acquisition for commonly kept reptile groups. Circles indicate mean mortality rate based on x 1000 bootstrap samples obtained from direct questions, lines represent 95% confidence intervals. Inset displays mean mortality rates for snakes, chelonians and lizards analysed separately for those reported to be captive bred (CB) and those reported to be either wild (W), or captive farmed (CF). Reptiles reported to be unknown origin (U) may represent captive bred or wild individuals and were therefore excluded. Refer to Table 2 for *n* numbers used in analysis of mortality rates.

Note that mean number of reptiles that died refers to the actual number not the mortality rate. Mortality rates incorporate the numbers of reptiles owned and are presented in Fig 1.

^a^ estimates for ‘all reptiles’ were derived post-data collection by combining ‘all snakes’, ‘all chelonians’ and ‘all lizards’ for individual respondents, therefore an aRRT response is not available for this category.

The combined estimated mortality rate for snakes, lizards and chelonians was 3.6% (Fig 1). Overall, lizards had higher mortality rates than chelonians and snakes. When split by groups, of the snakes, boas and pythons had the lowest mortality rates and king and rat snakes had the highest. Of the chelonians, tortoises and box turtles had lower mortality rates than turtles and terrapins, and of the lizards, iguanas had the lowest mortality rates whilst chameleons had the highest.

Data from four commercial operators analysed separately indicated a combined mortality rate of 0.7% for snakes, 1.1% for lizards and 0.03% for chelonians. This could not be bootstrapped due to the low sample size so error is not presented and the animals were kept for periods of between one week to two years for snakes (median = 8 weeks); one week to one year for chelonians (median = 2 weeks) and two weeks to 1.5 years for lizards (median = 3 weeks).

When mortality rates are explored in association with the source of the reptiles, there is an indication that captive bred reptiles have lower mean mortality rates than those of wild or captive farmed origin (Fig 1 inset). However, differences were not considered significant given overlapping confidence intervals.

There was a significant positive relationship between the perceived difficulty of keeping reptiles and the perceived survival rates, with those rated more difficult to keep also rated as having poorer survival rates (snakes: r_s_ = 0.25, n = 199, p <0.001; lizards: 0.42, n = 176, p <0.0001; chelonians: r_s_ = 0.47, n = 61, p <0.001). In addition, mortality rates reported via DQ were significantly and positively correlated with poorer perceived survival rates for snakes (r_s_ = 0.30, n = 199, p <0.001) and lizards (r_s_ = 0.26, n = 176, p <0.001); this relationship was not significant for chelonians (r_s_ = 0.06, n = 61, p = 0.63). There were no significant relationships between actual mortality rates reported via DQ and perceived difficulty in keeping different reptile groups (Table 3).

**Table 3:** Evaluation of respondents’ rating of ‘difficulty’ and ‘survival’ for different reptile groups, which they had acquired. Questions were asked using a five point Likert scale, with categories condensed for data presentation.

	Based on your experience and not preconceived ideas about the group, how easy or difficult is this group to keep?				Based on your experience and not preconceived ideas about the group, how do you rate the survival of this group in captivity?			
Taxa	*n*	Easy / very easy (%)	Neither easy nor difficult(%)	Very difficult / difficult (%)	*n*	Good / very good (%)	Neither good nor poor (%)	Very poor / poor (%)
**All reptiles**		NA	NA	NA		NA	NA	NA
**All snakes**	201	75.6	20.9	3.5	203	97.0	1.5	1.5
Boas & pythons	165	73.3	21.2	5.5	165	98.2	1.2	0.6
King & rat snakes	134	91.0	7.5	1.5	134	96.3	2.3	0.8
Other snakes	54	57.4	33.3	9.3	54	96.3	1.9	1.9
**All chelonians**	61	50.8	34.4	14.8	62	90.3	6.5	3.2
Tortoises & box turtles	49	63.3	26.5	10.2	49	93.9	2.0	4.1
Turtles & terrapins	17	64.7	23.5	11.8	18	88.9	11.1	0.0
**All lizards**	183	63.4	27.3	9.3	184	86.4	12.0	1.6
Chameleons	39	28.2	30.8	41.0	39	76.9	15.4	7.7
Geckoes	119	80.7	16.8	2.5	119	94.1	4.2	1.7
Skinks	18	61.1	27.8	11.1	18	88.9	11.1	0.0
Iguanas	22	45.5	18.2	36.4	22	72.7	22.7	4.6
Tegus & monitors	43	46.5	32.6	20.9	43	79.1	18.6	2.3
Agamids	83	62.0	17.0	4.0	83	90.4	9.6	0.0
Other lizards	20	65.0	15.0	20.0	20	95.0	0.0	5.0

### Evaluation of additive RRT

Respondents found aRRT easy to use with over 70% scoring it as ‘easy’ or ‘very easy’ to understand and only 9% scoring it as ‘difficult’ or ‘very difficult’. Over 56% of respondents felt that their answers were protected by aRRT compared to 13% who did not feel that their answers were protected. A large proportion (>58%) of respondents felt that the questions regarding mortality were ‘not very’ or ‘not at all’ sensitive (S1 Table).

There were no significant relationships between mortality rates (reported via DQ) and how sensitive people felt the questions regarding reptile mortality were: ‘all snakes’ (r_s_ = 0.12, n = 190, p = 0.09); chelonians (r_s_ = 0.12, n = 60, p = 0.34); lizards (r_s_ = 0.10, n = 172, p = 0.17). There were no significant relationships between reported mortality rates and how likely respondents felt people were to tell the truth for snakes (r_s_ = -0.12, n = 190, p = 0.11), chelonians (r_s_ = 0.08, n = 60, p = 0.52) and lizards (r_s_ = -0.03, n = 172, p = 0.72).

## Discussion

We estimated the overall mortality rate of pet reptiles (snakes, chelonians and lizards) amongst private breeders and keepers of reptiles, to be 3.6% within the first year of acquisition, which is considerably lower than some previous estimates. However, this rate varies amongst different reptile groups from 1.9% (boas and pythons) to 28.2% (chameleons). Additionally, there are indications that mortality rates are lower for captive bred individuals. As far as we know, this is the first survey to investigate reptile mortality rates amongst domestic consumers which also differentiates between commonly kept reptile groups. Our findings are intended to inform the ongoing debate concerning the regulation of the reptile trade both at national and international levels, and is also of conservation relevance when considering the implications of collecting reptiles from the wild. As governments and other stakeholders increasingly strive for an evidence base to inform policy, our findings may be seen as a robust mortality estimate for reptiles kept in the home by those who attend reptile shows.

### Mortality rates

Our study shows that among the commonly kept pet reptile groups, snakes had the lowest overall mortality rates in captivity, followed by chelonians, and then lizards. When this is compared with the wild, a comprehensive published study (based on a review of 20 species of snakes, 20 species of lizards and 17 species of turtles) reported annual adult survival rates to be lowest among lizards (~38% survival), followed by snakes (~64%), and then chelonians (~88%) [40]. If these survival rates are expressed as mortality rates then mortality in nature far exceeds our estimated mortality rates for reptiles in captivity. Whilst an understanding of the life histories of wild reptiles provides context and is useful to consider in relation to survival in captivity, wild and captive individuals are subject to somewhat different factors affecting their fitness, and therefore comparisons of wild and captive mortality rates should be made with caution. Additionally, in the example above, there are likely to be phylogenetic and geographical differences in the species studied. For example, the species composition of our dataset is representative of that in the home, which amongst the snakes, comprises a high proportion of boas and pythons. These are large bodied and long-lived, which may partly explain why we found snakes to have relatively low mortality rates. Indeed, according to a recent publication which collated longevity data, life history traits and environmental factors for 1,000 species of lizards and snakes (10% of the known species diversity), longevity in the wild is related to body size, brood frequency, age at first reproduction, predation pressure, environmental factors such as latitude and climate, and diet [41].

Whilst our estimates for mortality rates of most lizards were between 5% and 10%, chameleons had a higher mortality rate of 28%. Chameleons require specialised husbandry [42] and published reports on the longevity of this group in nature are limited. In the available studies, Cape dwarf chameleons (*Bradypodion pumilumare*) are reported to have annual survival rates of approximately 5% [43]; female panther chameleons (*Furcifer pardalis*) seldom live longer than one year, whilst males live longer [44] and studies have revealed particularly short post-hatching life spans of four to five months for Labord’s chameleon (*Furcifer labordi*) [45]. However, due to the paucity of research in this area it is difficult to draw solid conclusions about chameleon survival in the wild, and some species do have the capacity to reach ages of up to nine years in captivity [46]. In any case, specialism does not necessarily correspond with high mortality in captivity, as indicated by our finding that actual mortality rates were significantly correlated with perceived survival rates (high mortality, poor rated survival), but not with how difficult respondents felt the reptile groups were to keep. Difficulty keeping a reptile may therefore not always equate to high mortality, but may instead indicate higher requirements of husbandry and investment, which experienced keepers may be able to provide.

The majority of reptiles acquired over the previous five years were captive bred, and captive bred individuals appeared to have lower mortality rates. However, more data are required to thoroughly explore the difference in mortality rates between wild and captive bred individuals as this was just a non-significant trend. There are reports of wild reptiles in trade being sold as captive bred [47], and given that there may be some degree of sensitivity surrounding the topic, it can be difficult to verify their source. Differences between captive bred and wild individual mortality rates may arise from the fact that captive bred reptiles are thought to be easier to maintain in captivity, due to perceived lower aggression [2], lower levels of parasitic infection [2, 48] and easier acclimatisation to new conditions [48]. Whereas wild reptiles are subject to the additional stresses of capture in the wild, along with a potentially longer trade chain with more transit exchanges, which may in turn reduce the fitness of those animals. However, many captive bred individuals are also shipped internationally and little data exist on mortality of wild versus captive bred individuals along the trade chain or in captivity. A recent global review showed that the number of live, wild sourced reptiles (CITES Appendix II) in international commercial trade is decreasing whilst an increasing proportion appear to be sourced from more intensive systems such as ‘ranching’ (the rearing of young or eggs from the wild), and from countries where they do not exist naturally in the wild (i.e. captive bred) [6]. The implications of this are complex as in some circumstances and under the appropriate regulatory requirements, sustainable use of wild animals may contribute to conservation and livelihoods in developing countries where the species originate [49, 50]. However, there is currently little comparable information on the benefits and impacts of alternative production systems of pet reptiles, and analysis is complicated by reports that captive production and ranching systems are sometimes used to launder illegally wild caught animals [47] and can have negative impacts on wild populations [51].

It is important to consider that whilst respondents represented a range of experience levels and a wide catchment area in terms of postcode areas, they represent only a subset of reptile keepers in the UK, many of whom may not visit annual reptile shows. Additionally, the data presented here represent only one part of the trade chain, with mortality occurring at any stage of that chain before animals reach the home (e.g. during transit, wholesale, or in the pet shop), meaning the cumulative mortality may be much higher. Data concerning mortality all along the trade chain from source to consumer are scarce, but estimates during shipment and in retail suggest that average mortality rarely exceeds 4.5% at each stage [19, 20, 21, 22], apart from in some isolated cases [23, 26]. Additionally, data from four commercial operations in this study, which represent an additional stage prior to the reptiles reaching the final consumer, indicated low mortality rates of less than 1.2%, however as the reptiles were kept for varying durations they are not directly comparable to the annual mortality rates we present for private breeders and keepers of reptiles. More comprehensive and recent research at different points of the chain or by following specimens along the trade chain will allow greater understanding of overall mortality.

### Method comparison

We found no significant differences between mortality rates estimated via aRRT and DQ. Previous studies have reported that although RRT may improve data validity [52], the benefits of using such specialised questioning techniques decrease with decreasing topic sensitivity [30, 34]. Contrary to our beliefs when embarking upon this study, only 16% of respondents thought that the questions regarding reptile mortality were sensitive, which explains why there were no detectable differences between estimates achieved with the two methods. Accordingly, we have an increased level of confidence in the estimates obtained from using direct questions. The low level of sensitivity also explains why there were no significant correlations between reported mortality rates and how sensitive respondents found the questions, or how likely they felt people would be to tell the truth. Nonetheless, the majority of people (>70%) found aRRT easy to use and most felt that their answers were protected by the method suggesting that there is utility in the technique.

## Conclusion

Our research suggests that the number of reptiles that die in the home within one year of acquisition by private keepers and breeders of reptiles who attend reptile shows is relatively low (3.6%), and corresponds with a recent study conducted using an online questionnaire, which reported mortality rates of 3.25% [22]. However, some taxa evidently have higher mortality rates than others and may therefore be candidates for further research and targeted improvements regarding trade chain management and captive care requirements. Despite reporting a low mortality rate within the first year, mortality rates in the home after the first year are unlikely to be linear and are therefore not necessarily accumulative at the same rate. Additionally, we are unable to draw conclusions regarding specific welfare conditions of those reptile groups in captivity as this was not the purpose of this study. From a welfare perspective, and in order to add context, it may be interesting to compare our mortality rates with those of other commonly kept pet animals. Whilst there is limited data available, the only broadly comparable available study conservatively estimated that over a one year period (1996) in the US, 8.3% and 7.9% of cats and dogs died respectively [53].

An improved understanding of mortality rates of reptiles in the home may guide the regulation of the reptile pet trade and have direct policy implications. Whilst species may survive collection, breeding or transport, if they cannot be adequately maintained in captivity by end-users, then as long as demand exists for those animals, elevated numbers will be required to replace dead animals. In cases where species are harvested from the wild, this may directly impact species conservation where inadequate monitoring or sustainable use programs exist at the source. With improved understanding of reptile mortality, this can be taken into account when impact statements for traded species (“non-detriment findings”) are considered for species regulated under CITES. The EU Wildlife Trade Regulations, which implement CITES in the EU, contain a clause (Council Regulation (EC) No 338/97, Article 1.6) relating to 'live specimens of species listed in Annex B which have a high mortality rate during shipment or for which it has been established that they are unlikely to survive in captivity for a considerable proportion of their potential life span'. Under this clause, trade of reptiles shown to have high mortality rates in captivity could be suspended.

Whilst this study considers mortality of reptiles in the home, mortality may occur at various points in the trade chain and therefore the length and management of the supply chain is likely to be an important factor concerning overall survival. Cases of high mortality in the trade are reported [23, 26], but these cases do not appear to be frequent. Nevertheless, efforts must be made to prevent these. It remains to be seen whether certified trade chains could be feasible within the pet trade, in order to help understand and improve the process from supplier to consumer. This has the potential to increase transparency and consumer confidence in reptiles shipped cross globally, particularly in cases where wild trade supports sustainable use and conservation in developing countries.

## Supporting Information

S1 File Copy of questionnaire used in study. (PDF)

S2 File Additive RRT instruction cards. (PDF)

S3 File Additive RRT methodology further information. (PDF)

S1 Table Respondents’ evaluation of Additive RRT. Evaluation of additive RRT, including percentage responses for each category. Questions were asked according to a five point Likert scale, with categories condensed for data presentation.
(PDF)
