# Correction: Captive Reptile Mortality Rates in the Home and Implications for the Wildlife Trade

**DOI:** 10.1371/journal.pone.0157519

**Published:** 2016-06-06

**Authors:** Janine E. Robinson, Freya A. V. St. John, Richard A. Griffiths, David L. Roberts

The authors wish to add the following information to their article: Co-author Richard Griffiths serves as the President of the British Herpetological Society (BHS): http://www.thebhs.org.
