# Supplementary material for: Captive Reptile Mortality Rates in the Home and Implications for the Wildlife Trade
Source: PLoS One. 2015 Nov 10;10(11):e0141460. doi: 10.1371/journal.pone.0141460 (PMC4640569; doi:10.1371/journal.pone.0141460)
Supplement: S1 File — (PDF) [file pone.0141460.s001.pdf]

This questionnaire is not self-complete and should be administered by a trained research assistant

**Date:**		**Interviewer:**	
**Location:**			

REPTILE QUESTIONNAIRE

DICE University of Kent

**DO YOU GIVE YOUR CONSENT TO PARTICIPATE IN THIS STUDY?** ***TICK BOX***	☐

1	**Have you acquired a reptile in the last five years?** ***please circle***	**Y / N**

If yes, continue with questionnaire

2	**What type of reptile keeper do you consider yourself to be?**	***Tick one option***
a	Private reptile keeper (those keeping reptiles for pleasure)	
b	Private reptile breeder (those keeping and breeding reptiles mainly for pleasure)	
c	Private reptile breeder (those keeping reptiles mainly for monetary gain)	
d	Commercial enterprise (those buying/selling/breeding as part of a business)	

If Q2 d was selected, ask question 3. Otherwise, continue to Q4

3	**Do you sell dry goods as well as animals?** ***please circle***	**Y / N**

4	**What do you understand the following terms to mean?** ***Please state***	
	Captive Bred	
	Long Term Captive	
	Captive Farmed	
	Ranched	
	Wild Caught	

The following questions relate to your experience keeping different reptile groups. Anything you know as ‘Long Term Captive (LTC)’ is included with the ‘Wild’ category for this questionnaire. I am going to ask you questions for each reptile group and then split it down, so it may seem like I am asking the same question twice in some cases.

	**Have you acquired any ____________in the last five years?** This includes reptiles brought **into** collection, not births		5. **[RRT]** OF THE ________ THAT YOU ACQUIRED OVER THE LAST FIVE YEARS, HOW MANY DIED **WITHIN THE FIRST 12 MONTHS?**	6. What is the average amount of time you hold this group before rehoming/sale/ exchange, or do you not move them on?	7. How many years’ experience do you have in keeping this reptile group?	**Based on your experience and not preconceived ideas about the group**		10. How many ________have you acquired in the last **5** years, excluding births? If you are unsure, please estimate.	**22. [Direct Q] To be asked at end.** Of the _______ that you acquired over the last 5 years, how many died within the first 12 months?
						8. How easy or difficult is this group to keep? *[scale card]*	9. How do you rate the survival of this group in captivity? *[scale card]*		
	**SNAKES**	all							
	Snakes	C							
		W		GO TO Q8					
		CF							
		U							
	**Boas & pythons**	all							
	Boas & pythons	C							
		W							
		CF							
		U							
	**King & rat snakes**	all							
	King snakes (Lampropeltis) & rat snakes (Elaphe)	C							
		W							
		CF							
		U							
	**Other snakes**	all							
	Other snakes	C							
		W							
		CF							

			5. [RRT] OF THE _______ THAT YOU ACQUIRED OVER THE LAST FIVE YEARS, HOW MANY DIED **WITHIN THE FIRST 12 MONTHS?**	6. What is the average amount of time you hold this group before rehoming/sale/ exchange, or do you not move them on?	7. How many years' experience do you have in keeping this reptile group?	**Based on your experience and not preconceived ideas about the group** 8. How easy or difficult is this group to keep? *[scale card]*	9. How do you rate the survival of this group in captivity? *[scale card]*	10. How many _______have you acquired in the last **5** years, excluding births? If you are unsure, please estimate.	**22. [Direct Q] To be asked at end.** Of the ______ that you acquired over the last 5 years, how many died within the first 12 months?
	**Have you acquired any ___________in the last five years?** This includes reptiles brought **into** collection, not births	U							
	**CHELONIANS (tortoises etc)**	all							
	Chelonians (tortoises, turtles & terrapins)	C							
		W							
		CF							
		U							
	**Tortoises & box turtles**	all							
	Tortoises (Testudo) & box turtles (terrapene)	C							
		W							
		CF							
		U							
	**Terrapins & turtles**	all							
	Terrapins & turtles	C							
		W							
		CF							
		U							

	**Have you acquired any ___________in the last five years?** This includes reptiles brought **into** collection, not births		5. **[RRT]** OF THE _______ THAT YOU ACQUIRED OVER THE LAST FIVE YEARS, HOW MANY DIED **WITHIN THE FIRST 12 MONTHS?**	6. What is the average amount of time you hold this group before rehoming/sale/ exchange, or do you not move them on?	7. How many years' experience do you have in keeping this reptile group?	**Based on your experience and not preconceived ideas about the group.** 8. How easy or difficult is this group to keep? *[scale card]*	9. How do you rate the survival of this group in captivity? *[scale card]*	10. How many _______ have you acquired in the last **5** years, excluding births? If you are unsure, please estimate.	**22. [Direct Q] To be asked at end.** Of the animals that you acquired over the last 5 years, how many died within the first 12 months?
	**LIZARDS**	all							
	Lizards	C		→	→				
		W							
		CF							
		U							
	**Chameleons**	all							
	Chameleons	C							
		W							
		CF							
		U							
	**Geckos**	all							
	Geckos	C							
		W							
		CF							
		U							
	**Skinks**	all							
	Skinks	C							
		W							
		CF							
		U							

	**Have you acquired any ___________in the last five years?** This includes reptiles brought **into** collection, not births		5. **[RRT]** OF THE _______ THAT YOU ACQUIRED OVER THE LAST FIVE YEARS, HOW MANY DIED **WITHIN THE FIRST 12 MONTHS?**	6. What is the average amount of time you hold this group before rehoming/sale/ exchange, or do you not move them on?	7. How many years' experience do you have in keeping this reptile group?	**Based on your experience and not preconceived ideas about the group.**		10. How many _______ have you acquired in the last **5** years, excluding births? If you are unsure, please estimate.	**22. [Direct Q] To be asked at end.** Of the animals that you acquired over the last 5 years, how many died within the first 12 months?
						8. How easy or difficult is this group to keep? *[scale card]*	9. How do you rate the survival of this group in captivity? *[scale card]*		
	**Iguanas**	all							
	Iguanas	C							
		W							
		CF							
		U							
	**Tegus & monitors**	all							
	Tegus & monitors (varanus)	C							
		W							
		CF							
		U							
	**Agamids (dragons etc)**	all							
	Agamids (incl. water dragons & bearded dragons)	C							
		W							
		CF							
		U							
	**Other lizards**	all							
	Other lizards	C							
		W							
		CF							

	Have you acquired any ___________in the last five years? This includes reptiles brought **into** collection, not births		5. **[RRT]** OF THE _______ THAT YOU ACQUIRED OVER THE LAST FIVE YEARS, HOW MANY DIED **WITHIN THE FIRST 12 MONTHS?**	6. What is the average amount of time you hold this group before rehoming/sale/ exchange, or do you not move them on?	7. How many years' experience do you have in keeping this reptile group?	**Based on your experience and not preconceived ideas about the group.** 8. How easy or difficult is this group to keep? *[scale card]*	9. How do you rate the survival of this group in captivity? *[scale card]*	10. How many _______ have you acquired in the last **5** years, excluding births? If you are unsure, please estimate.	**22. [Direct Q] To be asked at end.** Of the animals that you acquired over the last 5 years, how many died within the first 12 months?
		U							

11	**How many years have you been keeping reptiles?** ***please circle***				
	0-1 years	2-5 years	6-10 years	11-20 years	21+ years

General questions

12	**Are you a member of any of the following groups or societies?**	***Tick all that apply***
	British Herpetological Society (BHS)	
	International Herpetological Society (IHS)	
	Local or regional society i.e. Thames & Chiltern Herpetological Group etc.	
	Taxa specific society e.g. British Chelonia Group	
	Other herpetological or conservation society (including ARG UK)	
	I am not a member of any herpetological or conservation groups or societies	

13	**Do you have any specific training or qualifications relating to reptile biology or care (informal or formal) e.g. college course, worked in zoo or lab?** ***please circle***	**Y / N**
	Please specify	

14	**Gender** ***please circle***	**M / F**

15	**Country of residence** ***please state***	

16	**First part of post code** ***please state***	

17	**What is your year of birth?** ***please state***	

18	**What is your marital status?** ***Please circle***		
	Single, never married	Married or domestic partnership	Widowed, divorced or separated

19	**What is the highest level of education you have completed?**	***Tick one option***
	GCSE/O-Level/CSE	
	Vocational qualifications (NVQ1+2)	
	A-Level or equivalent (NVQ3)	
	Bachelor or degree or equivalent (NVQ4)	
	Masters/PhD or equivalent	
	Other *(please specify)*	

	No formal qualifications	

20	**Employment/activity**	***Tick one option***
	Working full time (more than 30 hours a week)	
	Working part-time (0-30 hours a week)	
	Self-employed	
	Student *(skip Q21)*	
	Retired *(skip Q21)*	
	Temporarily unemployed and seeking work *(skip Q21)*	
	Permanently unemployed (e.g. chronically sick, independent means) *(skip Q21)*	
	Not in paid work for other reason (e.g. house wife, carer) *(skip Q21)*	

21	**What personal annual income band do you fall into (before tax)?** ***please circle***				
	Under £20,000	£20,001 - 30,000	£30,001 - 50,000	£50,001 - 70,000	Over £70,000

Q 22 – DIRECT QUESTION

Evaluation questions

23	**How easy or difficult did you find the card method to use?** ***(please circle)***				
	Very easy	Easy	Neither easy nor difficult	Difficult	Very difficult

24	**When using the card method, how protected or unprotected did do you feel your answers were? (*please circle)***				
	Very protected	Somewhat protected	Neither protected nor unprotected	Not very protected	Not at all protected

25	**How sensitive do you consider the questions about the quantity of your reptiles that have died in the first year of you owning them? (*please circle)***				
	Very sensitive	Somewhat sensitive	Neither sensitive nor insensitive	Not very sensitive	Not at all sensitive

26	**When asked directly, how likely do you think people would be to tell the truth when asked about the quantity of reptiles that have died in the first year? (*please circle)***				
	Very likely	Likely	Neither likely nor unlikely	Unlikely	Very likely

Thank you for taking the time to complete this questionnaire.
