# Supplementary material for: Captive Reptile Mortality Rates in the Home and Implications for the Wildlife Trade
Source: PLoS One. 2015 Nov 10;10(11):e0141460. doi: 10.1371/journal.pone.0141460 (PMC4640569; doi:10.1371/journal.pone.0141460)
Supplement: S2 File — (PDF) [file pone.0141460.s002.pdf]

There is no way I can trace your answers back to you. You remain completely anonymous.

Example

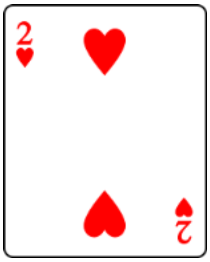

INSTRUCTIONS

Please pick a card from the pack – do not let me see what card you have

Remember the rules:

Queen = answer the question truthfully

Number card = add the number on the card you have picked to your true response and report the total (Ace = 1)

QUESTION: **Of the _ _ _ _ _ _ _ _ _ _ _ that you acquired over the last five years, how many died within the first 12 months?**

There is no way I can trace your answers back to you. You remain completely anonymous.

Example

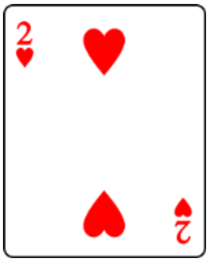

INSTRUCTIONS

Please pick a card from the pack – do not let me see what card you have

Remember the rules:

Queen = answer the question truthfully

Number card = add the number on the card you have picked to your true response and report the total (Ace = 1)

QUESTION: **Of the _ _ _ _ _ _ _ _ _ _ _ that you acquired over the last five years, how many died within the first 12 months?**
