# Supplementary material for: Captive Reptile Mortality Rates in the Home and Implications for the Wildlife Trade
Source: PLoS One. 2015 Nov 10;10(11):e0141460. doi: 10.1371/journal.pone.0141460 (PMC4640569; doi:10.1371/journal.pone.0141460)
Supplement: S3 File — (PDF) [file pone.0141460.s003.pdf]

S2 Appendix: Additive RRT Methodology further information

During model development, a number of models were conducted using bootstrapped artificial data (in which mean μ_x and variance σ^2_x were known), in order to determine the most appropriate model. This included deciding on the proportion of people who should be instructed to tell the truth (T), and the composition of numbers to make up the scrambling device (mean μ_s and variance σ^2_s). These factors were determined based on a combination of model efficiency (best predictive power and least error), and what would work practically in the field and lead to the least confusion amongst respondents. For example, in order to minimise variance associated with the model, according to the following formula for estimating variance [1], T should be >0.8 or <0.2:

$$Var(\mu_x) = \frac{\sigma_x^2}{n} + \frac{(1-T)(\sigma_x^2 + T\mu_s^2)}{n}$$

T > 0.8 would have required a high proportion (more than 80%) of respondents to answer the sensitive question truthfully which we felt would result in respondents feeling less protected than if less than 20% were required to answer truthfully.

In order to allow the best protection it was important to have an idea of the approximate range of responses that would be given by the respondents. As the numbers in the cards ranged from one to 10, respondents were most protected if their true responses were low numbers. For example, a respondent giving a reported response of one is completely protected: they may have had one reptile die and reported their true answer, or they may have had no reptiles die and picked a number card of one to report. The same goes for respondents reporting answers between one and 10. However, if a respondent gives a reported response of 11, then the interviewee know that they have had at least one reptile die, but they do not know the exact number as any number card between one and 10 may

have been added to their true value. Presuming that the question is sensitive, this means that although their exact true answer is still masked, this person has slightly less protection as one element of their true response (the fact that at least one animal died) is revealed. If you get to a reported response of 20, you know that at least 10 of their reptiles had died. Therefore, the level of protection the respondent is afforded, decreases as the upper range of true responses increases. Conversely, if a respondent reported a response of zero, the interviewee knows that the respondent has not had any reptiles die at all, however this was not considered to be a sensitive scenario.

It was difficult to know the number of reptiles' people may report owning and the expected mortality rates of those reptiles, as there is little primary data in this area. However, we used a recent study based on online questionnaires with a reported mortality rate of 3.3% [2], and informal discussions with reptile keepers to inform our model choice. We also decided to exclude commercial operations from the analysis, as they are likely to have owned a much larger numbers of reptiles and therefore fit less well with our model.

Following data collection we tested for a relationship between the number of reptiles respondents reported owning and how protected those respondents' felt when using the aRRT. There were no significant correlations for snakes (r_s = 0.08, n = 194, p = 0.24), lizards (r_s = 0.07, n = 179, p = 0.32) or chelonians (r_s= -0.17, n = 60, 0 = 0.19), suggesting that the concerns referred to above regarding those with larger numbers of reptiles not feeling as much protection by the model, did not appear to be an issue in this case.

References

1. Sehra S. Two-stage Optional Randomized Response Models: ProQuest; 2008.

2. Clark B. A report looking at the reptile keeping hobby, those who want it banned and why? 2013.
