# Supplementary material for: Captive Reptile Mortality Rates in the Home and Implications for the Wildlife Trade
Source: PLoS One. 2015 Nov 10;10(11):e0141460. doi: 10.1371/journal.pone.0141460 (PMC4640569; doi:10.1371/journal.pone.0141460)
Supplement: S1 Table — Evaluation of additive RRT, including percentage responses for each category. Questions were asked according to a five point Likert scale, with categories condensed for data presentation. (PDF) [file pone.0141460.s004.pdf]

S1 Table. Respondents' evaluation of Additive RRT. Evaluation of additive RRT, including percentage responses for each category. Questions were asked according to a five point Likert scale, with categories condensed for data presentation.

Questions	Responses		
How easy or difficult did you find the card method to use?	Very easy / easy<br>**73.6% (n = 190)**	Neither easy nor difficult<br>**17.4% (n = 45)**	Difficult / very difficult<br>**8.9% (n = 23)**
When using the card method, how protected or unprotected did you feel your answers were?	Very protected / Somewhat protected<br>**56.5% (n = 143)**	Neither protected nor unprotected<br>**30.4% (n = 77)**	Not very protected / not at all protected<br>**13.0% (n = 33)**
How sensitive do you consider the questions about the quantity of your reptiles that have died in the first year of you owning them?	Very sensitive / somewhat sensitive<br>**15.9% (n = 41**	Neither sensitive nor insensitive<br>**26.0% (n = 67)**	Not very sensitive / not at all sensitive<br>**58.1% (n = 150)**
When asked directly, how likely do you think people would be to tell the truth when asked about the quantity of reptiles that have died in the first year?	Very likely / likely<br>**41.2% (n = 105)**	Neither likely nor unlikely<br>**25.5% (n = 65)**	Unlikely / very unlikely<br>**33.3% (n = 85)**
